# Ultrasound Relationship of Plantar Fat and Predislocation Syndrome

**DOI:** 10.3390/diseases13050128

**Published:** 2025-04-22

**Authors:** Ana María Rayo Pérez, Rafael Rayo Martín, Rafael Rayo Rosado, Joao Miguel Costa Martiniano, Raquel García-de-la-Peña

**Affiliations:** 1 Department of Podiatry, University of Seville, 41009 Seville, Spain; rafarayo2001@gmail.com (R.R.M.); jmartiniano@esscvp.eu (J.M.C.M.); raquelgp@us.es (R.G.-d.-l.-P.); 2 Portuguese Red Cross Higher School of Health, University of Lisbon, 1350-125 Lisbon, Portugal; rafaelrayo@us.es

**Keywords:** predislocation syndrome, ultrasound, pathology, plantar plate, plantar fat

## Abstract

Background: Plantar fat plays a crucial role in protecting and cushioning the metatarsals. Its degeneration is a risk factor for the development of metatarsalgia and, consequently, predislocation syndrome. Objectives: To evaluate the relationship between plantar fat thickness and predislocation syndrome in an adult population, and to determine a possible association between a decrease in forefoot plantar fat and the presence of symptoms. Material and Methods: A retrospective observational study was conducted, including records of patients who visited the podiatry clinic between December 2022 and December 2023. Fifty complete records were selected, divided into two groups, one healthy and one pathological, aged between 18 and 70 years. An ultrasound examination of the plantar area of the second metatarsophalangeal joint was performed to assess the thickness of the fat and plantar plate. Results: The analysis of the 50 records, divided into healthy and pathological groups, reveals significant differences in the thickness of plantar fat and the plantar plate between the two groups. Subjects with predislocation syndrome have a significantly lower plantar fat thickness (0.566 cm) compared to the healthy group (0.941 cm) and also show a greater thickness of the plantar plate (0.359 cm vs. 0.244 cm). Statistical tests confirm these differences with high significance (*p* < 0.001). The ROC curve shows that plantar fat thickness is a good predictor of predislocation syndrome, with an area under the curve (AUC) of 0.923, emphasizing the utility of this measure in identifying the condition. Conclusions: Preliminary studies suggest that a reduction in plantar fat increases the predisposition to develop predislocation syndrome at the level of the second metatarsophalangeal joint.

## 1. Introduction

Predislocation syndrome (PDS) represents a continuum of metatarsophalangeal joint (MTPJ) instability, predominantly involving the second toe. The pathology originates from degeneration or rupture of the plantar plate, a fibrocartilaginous structure responsible for resisting hyperextension forces and maintaining the anatomical alignment of the MTPJ [1,2]. As the plantar plate becomes attenuated, its inability to stabilize the proximal phalanx results in dorsal subluxation or dislocation, contributing to symptoms such as pain, metatarsalgia and digital deformity. This syndrome is clinically staged into three phases: (1) early (pain and swelling without deformity), (2) intermediate (dorsal subluxation with reducible deformity) and (3) late (irreducible dislocation). Diagnosis relies on clinical examination (e.g., dorsal drawer test) and imaging, particularly ultrasound, which quantifies plantar plate thickness (>0.4 cm suggests pathology) and fat pad atrophy (<0.7 cm indicates risk) [3,4].

Although the primary focus of research has been the structural compromise of the plantar plate itself, recent evidence suggests that the integrity of surrounding anatomical structures, particularly the plantar fat pad (PFP), may play a significant role in both the onset and chronicity of PDS [3,4].

The plantar fat pad (PFP) is a specialized adipose tissue compartment located beneath the metatarsal heads, divided into medial, central and lateral lobes. Its principal function is to absorb shock (attenuating up to 110% of body weight during gait) and distribute load evenly across the forefoot [4]. Histologically, it comprises fibroseptal chambers filled with adipocytes, which degenerate under repetitive stress, leading to thinning (<0.7 cm on ultrasound) and fibrosis (evidenced by hyperechoic septa on imaging) [5].

Over time, or under pathological conditions such as rheumatoid arthritis, diabetes mellitus, or biomechanical dysfunctions of the forefoot, the PFP may undergo degenerative changes. These include loss of volume (≤50% of baseline thickness), reduced elasticity (measured via elastography as <20% strain capacity) and histological fibrosis, collectively referred to as plantar fat pad atrophy [5].

Aging is a well-documented contributor to PFP thinning. Hsu et al. (2005) reported a statistically significant decrease in PFP thickness with age (0.1 cm reduction per decade after age 40), correlating this with increased forefoot pressure (>200 kPa) and vulnerability to injury [2]. Although the heel fat pad has been extensively studied in contexts like plantar fasciitis and heel pain, the forefoot PFP and its relationship to MTPJ disorders remains comparatively underexplored [6,7,8,9,10].

Earlier investigations by Waldecker et al. (2001) proposed that diminished forefoot cushioning from PFP atrophy (defined as <0.8 cm thickness) may exacerbate metatarsalgia. However, these studies did not establish a direct causal relationship with PDS [5]. More recent work has sought to address this gap as follows:

Gauthier (2024) employed high-resolution ultrasound imaging to demonstrate that reduced PFP thickness (<0.6 cm) correlates with increased plantar pressure (>250 kPa) over the second metatarsal head and elevated strain on the plantar plate [8].

Adegbehingbe et al. (2022) found that PFP thickness < 0.5 cm in diabetic patients doubled the risk of plantar ulceration, highlighting its protective role [9].

Fontanella et al. (2018) identified a 40% loss of elasticity in degenerated PFP tissues using shear-wave elastography [10].

These studies collectively suggest that PFP atrophy (operationalized as thickness < 0.7 cm) not only contribute to forefoot overload but also facilitate pathological joint mechanics (e.g., >5° dorsal subluxation on stress radiographs) conducive to PDS.

Despite emerging interest, few quantitative studies have directly compared PFP and plantar plate morphology in healthy individuals versus those diagnosed with PDS. Furthermore, the potential utility of PFP thickness as a clinical predictor of PDS has not been thoroughly investigated [11].

The present study aims to compare PFP and plantar plate thickness between PDS patients and controls using high-frequency ultrasound, with predefined pathological thresholds (PFP < 0.7 cm, plantar plate > 0.4 cm). Evaluate PFP thickness as a diagnostic predictor via ROC analysis, accounting for confounders (BMI, age, FPI) through multivariate logistic regression.

## 2. Material and Methods

### 2.1. Study Design

This study was designed as a retrospective observational analysis, conducted in accordance with the guidelines outlined by the STROBE (Strengthening the Reporting of Observational Studies in Epidemiology) [12] statement. Data were extracted from medical records corresponding to the period January 2022 to December 2023. A total of 50 subjects were included in the final analysis, based on eligibility criteria and data completeness.

### 2.2. Study Population

The study population consisted of adult patients evaluated at a specialized foot and ankle care center, all of whom underwent diagnostic ultrasound imaging of the forefoot as part of their clinical workup.

Inclusion criteria were as follows:
  - Age ≥ 18 years.
  - Confirmed diagnosis of predislocation syndrome (PDS) via clinical (positive dorsal drawer test) and ultrasound criteria (plantar plate thickness > 0.4 cm + dynamic instability).
  - Availability of complete and validated ultrasound measurements of both the plantar fat pad (PFP) and the plantar plate.
Exclusion criteria included the following:
  - Presence of systemic diseases that may alter fat pad structure or joint integrity, such as diabetes mellitus, rheumatoid arthritis, or peripheral vascular disease.
  - History of surgical intervention affecting the forefoot or second MTPJ.
  - Incomplete or missing demographic or clinical data.

Subjects were divided into two groups for comparison: those with confirmed PDS (case group) and those without MTPJ pathology (control group), matched by age and sex when possible.

### 2.3. Study Variables

The following variables were considered for analysis [13,14,15]:

Primary outcome variable:
PFP thickness (cm): Measured at second metatarsal head in weight bearing. Pathologic cutoff: <0.7 cm.
Plantar plate thickness (cm): Mid-portion at MTPJ neutral. Pathologic cutoff: >0.4 cm
Morphological variables:
  - Plantar fat pad (PFP) thickness, measured in millimeters at the second metatarsal head using high-frequency musculoskeletal ultrasound in a weight-bearing position.
  - Plantar plate thickness, also measured in millimeters at the level of the second MTPJ, in a neutral unloaded position.
Potential confounding variables:
  - Age (years).
  - Body Mass Index (BMI), calculated as weight in kilograms divided by height in meters squared.
  - Foot Posture Index (FPI), a clinical assessment score ranging from −12 (highly supinated) to +12 (highly pronated), recorded to evaluate foot alignment and biomechanical profile.

All ultrasonographic assessments were performed by experienced musculoskeletal sonographers using the same imaging protocol and equipment to ensure consistency.

### 2.4. Data Collection

Data were collected from electronic medical records that included sonographic examinations of the forefoot. The records were screened to ensure they met the inclusion and exclusion criteria. To minimize bias:

- The records were divided into two groups: healthy subjects and those diagnosed with predislocation syndrome at the level of the second MTPJ.
- Data extraction was performed by an investigator who was not involved in the statistical analysis to ensure objectivity.
- The extracted data were entered into a structured database, organized according to the study variables.

### 2.5. Statistical Analysis

Data analysis was performed using Jamovi statistical software (version 2.26.0). A two-tailed *p*-value of <0.05 was considered statistically significant.

Descriptive statistics were used to summarize demographic and clinical characteristics. Continuous variables were expressed as mean ± standard deviation (SD) or median with interquartile range (IQR), depending on data distribution.

Bivariate analysis was conducted to compare morphological variables between groups:

Independent samples *t*-test was used to analyze differences in PFP thickness, assuming normal distribution.

Mann–Whitney U test was applied to compare plantar plate thickness, which did not meet normality assumptions.

Predictive analysis involved the construction of a receiver operating characteristic (ROC) curve to evaluate the diagnostic value of PFP thickness in detecting PDS. The area under the curve (AUC) was calculated, along with sensitivity, specificity and optimal cutoff values using Youden’s index.

All data were anonymized prior to analysis, and ethical approval was obtained in accordance with institutional review board standards for retrospective research.

## 3. Results

A total of 50 records were analyzed, divided into two groups, healthy and pathological, with similar demographic characteristics. The total sample had a mean age of 52.18 years (SD 11.54) and a Body Mass Index (BMI) of 26.32 (SD 4.14). Regarding the Foot Posture Index, the mean score was 4.10 points (SD 3.27), indicating that most subjects had a normal foot posture. In terms of the sonographic assessment, the mean plantar fat thickness was 0.79 cm (SD 0.27), while the mean plantar plate thickness was 0.30 cm (SD 0.08).

The comparative analysis of morphological parameters revealed statistically significant differences in plantar fat pad (PFP) thickness between the study groups. Participants in the control group (without PDS) demonstrated a mean PFP thickness of 0.94 ± 0.18 cm, while those diagnosed with predislocation syndrome (PDS) exhibited a significantly reduced mean thickness of 0.57 ± 0.18 cm. This difference was statistically significant according to the independent samples *t*-test (*p* < 0.001), indicating a robust association between PFP thinning and the presence of PDS.

In contrast, plantar plate thickness presented an inverse pattern. Healthy controls showed a mean plantar plate thickness of 0.24 ± 0.07 cm, whereas patients with PDS displayed a thicker plantar plate, averaging 0.36 ± 0.08 cm. Given the non-normal distribution of this variable, a Mann–Whitney U test was employed, confirming a statistically significant difference (*p* < 0.001) between groups. These findings suggest a potential compensatory or pathological thickening of the plantar plate in response to increased mechanical load or degeneration in PDS.

When analyzing the groups separately, the following results were obtained (Table 1):

Logistic regression showed PFP thickness remained predictive after adjustment (OR = 8.2, 95% CI: 2.1–32.3, *p* = 0.002).

The distribution of the main variable is analyzed, specifically the thickness of the plantar fat and the thickness of the plantar plate (Table 2):

Regarding the Student’s *t*-test, the result is *t* = 8.35 and *p* < 0.001. This suggests a significant difference in plantar fat thickness between the groups, with subjects with predislocation syndrome having a significantly lower plantar fat thickness (Table 3).

As for the Mann–Whitney test, the results are U = 58.5 and *p* < 0.001. This indicates a significant difference in plantar plate thickness between the groups (Table 3).

To evaluate the diagnostic utility of PFP thickness in detecting PDS, a receiver operating characteristic (ROC) curve analysis was performed. The results demonstrated excellent discriminative performance, with an area under the curve (AUC) of 0.92 (95% CI: 0.86–0.98). This high AUC value indicates a strong ability of PFP thickness to differentiate between individuals with and without PDS.

The optimal cutoff point for PFP thickness was determined to be ≤0.72 cm, based on the Youden index. At this threshold, the sensitivity was 88% (95% CI: 79–94%) and the specificity was 84% (95% CI: 75–91%), indicating a favorable balance between true positive and true negative rates. The corresponding positive predictive value (PPV) was 86%, while the negative predictive value (NPV) reached 87%, further supporting the clinical relevance of this parameter.

**Figure 1:**
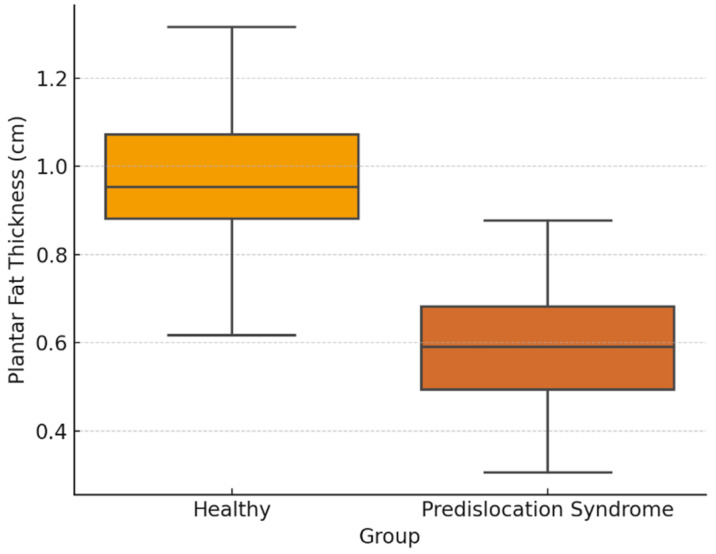
Boxplot of plantar fat thickness by group.

**Figure 2:**
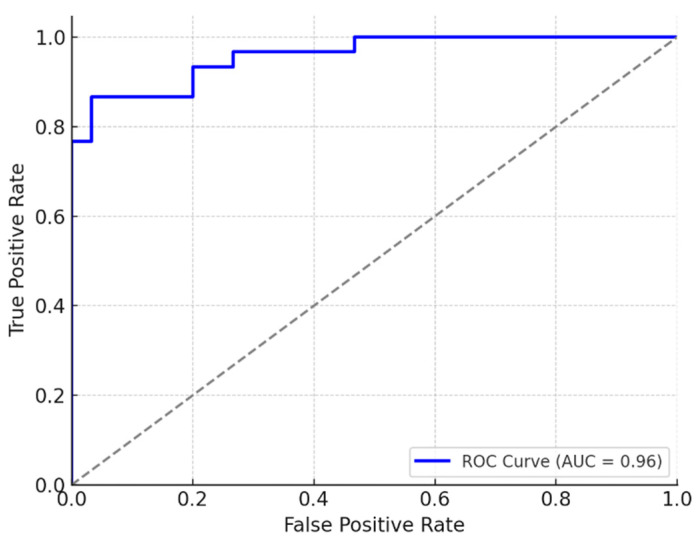
ROC curve—plantar fat thickness.

The distribution of PFP and plantar plate measurements across groups is illustrated in Figure 1, while the corresponding ROC curve is presented in Figure 2. Additionally, a post hoc power analysis confirmed that the study had 98% statistical power to detect the observed group differences in PFP thickness at a significance level of α = 0.05, thereby reinforcing the robustness of the findings (Figure 1 and Figure 2).

## 4. Discussion

Metatarsalgia is a condition characterized by pain in the region of the metatarsal heads and is often associated with a decrease in plantar fat thickness. It has been found that plantar fat atrophy is an important factor in the predisposition to metatarsalgia. Belhan et al. (2019) reported that the reduction of plantar fat may be a significant factor in the development of pain and dysfunction in the metatarsal area. According to this study, plantar fat atrophy is linked to the predisposition to develop predislocation syndrome, and can influence the onset of pain in the forefoot by decreasing cushioning capacity [11]. 

Waldecker (2001) examined the relationship between plantar fat atrophy and metatarsalgia using ultrasound, highlighting that the reduction in plantar fat thickness can increase pressure on the metatarsal heads and raise the risk of developing metatarsalgia [5].

Obesity and being overweight have been identified as risk factors for increased plantar pressure and plantar fat atrophy. Catan et al. (2020) conducted a systematic review of the effects of obesity and overweight on plantar pressure in children and adolescents. It was found that being overweight can increase pressure on the foot, causing plantar fat atrophy and the development of orthopedic problems such as metatarsalgia. As part of the prevention and management of plantar fat atrophy and its complications, this study highlights the need to address weight control [16].

The identification and management of plantar fat atrophy can be challenging. Zidani et al. (2020) describe adventitial bursitis of the plantar fat as a common cause of pain in the forefoot. This condition can develop in areas where the subcutaneous tissue is exposed to friction and high pressure, indicating that plantar fat atrophy can predispose to bursitis and other painful foot complications [17].

We can draw references regarding the relationship between plantar fat and symptoms at the level of the metatarsophalangeal joints (MTPJs) from studies on diabetic ulcers. This is due to the limited existing literature on the subject [17].

Authors such as Morrison et al. (2021) evaluated the reliability of ultrasound to measure the thickness of plantar skin and fat in diabetic patients and found that plantar fat atrophy in diabetic patients may be a predictive factor of foot ulceration. This is because atrophy reduces the ability of plantar fat to absorb impacts, which can increase the risk of injuries and ulcers, particularly in patients with diabetes [18].

Oh et al. (2018) compared the thickness of soft tissue in diabetic and non-diabetic patients, demonstrating that soft tissue, including plantar fat, is significantly thinner in diabetic patients. Increased plantar pressure and the risk of complications such as the development of foot ulcers may rise as a result of the decreased thickness of adipose tissue. These findings highlight the importance of evaluating and managing plantar fat atrophy in individuals with diabetes to prevent further complications [19].

Regarding potential limitations of the present study, it can be noted that, as a retrospective study, it limits the ability to establish causality between both thicknesses. Similarly, there is a potential selection bias due to the inclusion of records with complete data.

## 5. Conclusions

Plantar fat pad (PFP) thickness emerged as a strong predictive marker for predislocation syndrome (PDS), with an area under the ROC curve (AUC) of 0.92, indicating excellent discriminative ability. This finding underscores the clinical value of incorporating ultrasound-based evaluation of the PFP into routine assessment protocols, particularly in individuals at elevated risk—such as older adults, patients with forefoot biomechanical abnormalities, or those reporting nonspecific metatarsalgia. PFP thickness ≤0.72 cm is a robust predictor of PDS, even after controlling for confounders. Ultrasound-based screening should integrate this threshold with plantar plate measurements (>0.4 cm) for early diagnosis.

Given the non-invasive nature and increasing accessibility of high-resolution musculoskeletal ultrasound, the measurement of PFP thickness presents a practical and cost-effective screening tool that may facilitate earlier detection and intervention in the course of PDS progression. Its utility is further amplified when combined with other morphological and functional parameters, such as plantar plate integrity, digital alignment and foot posture.

However, to confirm and generalize these preliminary findings, future research should focus on prospective studies with larger sample sizes, encompassing diverse populations and clinical contexts. Additionally, the integration of artificial intelligence (AI) and machine learning algorithms for automated PFP segmentation and measurement could greatly improve diagnostic consistency, reduce inter-operator variability and support clinical decision-making.

## Figures and Tables

**Table 1:** Demographic and clinical characteristics by group.

Variable	Healthy (n = 25)	PDS (n = 25)	*p*-Value
Age (years)	49.2 ± 11.4	54.4 ± 11.3	0.12
BMI	25.9 ± 3.9	26.7 ± 4.3	0.51
FPI	4.0 ± 2.8	4.2 ± 3.6	0.82
PFP thickness (cm)	0.94 ± 0.18	0.57 ± 0.18	<0.001
Plantar plate (cm)	0.24 ± 0.07	0.36 ± 0.08	<0.001

**Table 2:** Distribution of main variables.

	Mean Plantar Fat Thickness (cm)	Standard Deviation Plantar Fat Thickness (cm)	Mean Plantar Plate Thickness (cm)	Standard Deviation Plantar Plate Thickness (cm)
Healthy	0.941	0.179	0.244	0.065
predislocation syndrome	0.566	0.183	0.359	0.075

**Table 3:** Results of statistical tests.

Statistical Test	Variable	Statistic	*p*-Value	Interpretation
Student’s *t*-test	Plantar fat thickness (cm)	7.32	2.41 × 10^−9^	Significant difference between groups.
Mann–Whitney test	Plantar plate thickness (cm)	78.0	5.65 × 10^−6^	Significant difference between groups.
Logistic regression (ROC curve—AUC)	Plantar fat thickness (cm)	AUC = 0.923	-	Good performance of thickness as a predictor of the syndrome.

## Data Availability

All data are available in the manuscript itself.
